# *Portulaca oleracea* L. extracts alleviate 2,4-dinitrochlorobenzene-induced atopic dermatitis in mice

**DOI:** 10.3389/fnut.2022.986943

**Published:** 2022-08-16

**Authors:** Wei-jie Lv, Jie-yi Huang, Shu-peng Li, Xiao-pei Gong, Jing-bo Sun, Wei Mao, Shi-ning Guo

**Affiliations:** ^1^College of Veterinary Medicine, South China Agricultural University, State Key Laboratory of Dampness Syndrome of Chinese Medicine, Guangzhou, China; ^2^State Key Laboratory of Dampness Syndrome of Chinese Medicine, The Second Affiliated Hospital of Guangzhou University of Chinese Medicine, South China Agricultural University, Guangzhou, China; ^3^Guangdong Technology Research Center for Traditional Chinese Veterinary Medicine and Natural Medicine, Guangzhou, China; ^4^Guangdong Provincial Key Laboratory of Prevention and Control for Severe Clinical Animal Diseases, Guangzhou, China; ^5^International Institute of Traditional Chinese Veterinary Medicine, Guangzhou, China

**Keywords:** atopic dermatitis, *Portulaca oleracea* L., immunomodulation, anti-inflammatory, anti-pruritic, 2,4-dinitrochlorobenzene (DNCB)

## Abstract

Atopic dermatitis (AD) is a common chronic allergic skin disease characterized clinically by severe skin lesions and pruritus. *Portulaca oleracea* L. (PO) is a resourceful plant with homologous properties in medicine and food. In this study, we used two different methods to extract PO, and compared the therapeutic effects of PO aqueous extract (POAE) and PO ultrasound-assisted ethanol extract (POEE) on 2,4-dinitrochlorobenzene (DNCB)-induced AD mice. The results showed that in POAE and POEE, the extraction rates of polysaccharides were 16.95% and 9.85%, while the extraction rates of total flavonoids were 3.15% and 3.25%, respectively. Compared with AD mice, clinical symptoms such as erythema, edema, dryness and ulceration in the back and left ear were alleviated, and pruritus behavior was reduced after POAE and POEE treatments. The thickness of the skin epidermis was thinned, the density of skin nerve fibers labeled with protein gene product 9.5 (PGP9.5) was decreased, and mast cell infiltration was reduced. There was a decrease in blood lymphocytes, eosinophils and basophils, a significant decrease in spleen index and a noticeable decrease in serum immunoglobulin E (Ig E). POEE significantly reduced the concentration of the skin pruritic factor interleukin (Il)-31. POAE and POEE reduced the concentration of skin histamine (His), down-regulated mRNA expression levels of interferon-γ (Ifnγ), tumor necrosis factor-α (Tnf-α), thymic stromal lymphopoietin (Tslp) and Il-4, with an increase of Filaggrin (Flg) and Loricrin (Lor) in skin lesions. These results suggested that POAE and POEE may inhibit atopic response and alleviate the clinical symptoms of AD by inhibiting the expression of immune cells, inflammatory mediators and cytokines. PO may be a potential effective drug for AD-like diseases.

## Introduction

Atopic dermatitis (AD) is a common, chronic, inflammatory skin disease, which is often accompanied by severe pruritus and high recurrence rate. Children have the highest incidence and are prone to relapse in adulthood. According to an extensive epidemiological survey, the prevalence rate is about 15–30% for children and 2–10% for adults around the world (1). In recent years, the incidence of AD shows a rising trend (2).

The clinical symptoms of AD include severe pruritus, impaired skin barrier, edema, erythema, dryness, ulcers, etc. In addition, AD is a primary immune abnormality, with elevated serum immunoglobulin E (Ig E) and immune cell infiltration. Because of the obvious appearance of the lesions, the tendency of the disease to recur, and the high cost of long-term treatment, the quality of work and life of AD patients are seriously affected, and even their emotions are inevitably disturbed (3). Therefore, it is of great significance to find some potential therapeutic agents for AD with low economic burden and effective in relieving dermatitis symptoms.

The pathogenesis of AD is not completely clear. Studies have shown that AD is driven by defects in terminal keratin-forming cell differentiation and strong type 2 immune responses (4). Currently, clinical medications used to treat AD include basic moisturizing creams, external application therapies, vitamin D supplements, topical corticosteroids, oral anti-inflammatory and antihistamines (5, 6). Steroids and calcineurin inhibitors (cyclosporine, tacrolimus) are still the first choice of drugs in acute attacks of AD (7). However, there is still a large unmet need for novel therapeutic approaches as these drugs have serious side effects, including adrenal failure, skin atrophy, neurotoxicity, nephrotoxicity and skin canceration (8).

Herbs have been reported to improve the severity of symptoms such as skin lesions and pruritus in AD (9). Clinical studies have found that the use of herbal medicines, such as Xiao-Feng-San, *Glycyrrhiza uralensis* Fisch., and *Lonicera japonica* Thunb., reduces the frequency of corticosteroid use and decreases exposure to corticosteroids in children with AD (10). Given the heterogeneity of the disease and the limitations of studies, more research is needed to demonstrate the effectiveness of herbs for AD. *Portulaca oleracea* L. (PO), which is called longevity vegetable in folklore, a medicinal food homolog (11). External use of PO for treating skin injuries and dermatitis has also been reported extensively. In general, the role of PO is to stimulate the angiogenesis of injured skin, regulate the proliferation of skin fibroblasts, promote the production of collagen fibers in the skin, and accelerate wound healing in the skin (12, 13). Previous studies on the active ingredients have revealed that polysaccharides and flavonoids of PO play important roles in the treatment of various diseases (14). However, the underlying mechanisms are still unclear.

In this study, the aqueous and ultrasound-assisted ethanolic extracts of PO (POAE and POAE) were used to compare the extraction rates of polysaccharides and total flavonoids. As well, we investigated the therapeutic effect of external application of PO on mice with AD-like lesions.

## Materials and methods

### Drug preparation

#### PO aqueous extract

PO (30 g), purchased from Guangzhou Nanbei Traditional Chinese Medicine Decoction Pieces Co., Ltd. (China), was first soaked in 300 mL of distilled water for 30 min. Then heated up to 180°C and maintained at 80°C for 30 min and filtered out the first extract. Repeat the above steps with another 300 mL of distilled water. Mix the extracts obtained. After concentrated to 30 mL by rotary evaporator, the aqueous extract of PO with the concentration of 1 g/mL was obtained and stored at 4°C. Our preliminary study found that 1 mg/mL POAE was more effective than 0.5 mg/mL POAE, so we chose this concentration as the PO extract concentration (Supplementary Figure 1).

#### PO ultrasound-assisted ethanol extract

PO (30 g) were broken to pieces and sieved through 60 pieces of mesh, dissolved in 600 mL of 70% ethanol, and extracted with ultrasound at 50°C for 40 min. The extract was filtered, and the upper layer of the solution was centrifuged and collected. After concentrated to 30 mL by rotary evaporator, the ethanol extract of PO with the concentration of 1 g/mL was obtained and stored at 4°C.

#### Hydrocortisone butyrate cream

0.1% hydrocortisone butyrate cream (HBC) was purchased from Shubang Pharmaceutical Co., Ltd. (China), as a positive control drug in this study.

### Determination of polysaccharide and total flavonoid contents

The concentrations of polysaccharides in POAE and POEE were measured using a multimode reader (EnSight, United States) at a wavelength of 490 nm, compared with glucose. The absorbance was measured at 510 nm and compared with rutin to calculate the total flavonoid concentration in POAE and POEE. Polysaccharide and total flavonoid extraction rates were expressed as a percentage of polysaccharide and total flavonoid content and PO raw material mass. Anhydrous glucose standard (NO. MO309BS) purchased from Dalian Meilun Biotechnology Co., Ltd. (China), and rutin standard (NO. PRF21071301) was purchased from Chengdu Biopurify Phytochemicals Co., Ltd. (China).

### Animals

Following AAALAC guidelines, all animal experimental procedures comply with the standards of the South China Agricultural University Experimental Animal Ethics Committee. The animal experimental procedures were approved by the Ethics Committee.

Six-week-old KM male mice, weighing 30 ± 2 g, purchased from the Experimental Animal Management Center of Southern Medical University. Animals were kept in the South China Agricultural University Laboratory Animal Center (SYXK 2019-0136) at a room temperature of 25 ± 2°C and relative humidity of 55 ± 5%. Mice were free to feed and drink.

### Sensitization and treatment of AD mice

After 7 days of adaptation, all mice were shaved on the back (2.5 cm × 2.5 cm). The mice were randomly divided into 5 groups: Control, DNCB, HBC, POAE, POEE groups, 5 mice in each group.

Sensitization: 2% DNCB and 0.5% DNCB dissolved in a mixture of acetone and olive oil (3:1 v/v) (15, 16). 2% DNCB was challenged on the dorsal skin (200 μL) and left ear (100 μL) of the DNCB, HBC, POAE and POEE groups, followed by 0.5% DNCB every two days starting on day 3 for 4 weeks. Equal volumes of acetone and olive oil mixture were used as controls in Control group.

Intervention: Starting from the second week (day 8), each group of mice was given external drug intervention on the dorsal skin twice a day for 3 weeks (until day 28). POAE group was treated with 3 mL 1 g/mL POAE, POEE group was treated with 3 mL 1 g/mL POEE, HBC group was treated with 1 g 0.1% HBC, while Control and DNCB group was treated with 3 mL 0.9% normal saline (NS). At the end of the animal experiments, samples were collected as needed.

### Clinical symptoms and SCORing of atopic dermatitis

Mental status, activity and mortality were observed and recorded for each group of mice. According to a previous study, dorsal skin severity scores were recorded weekly for AD mice based on four skin symptoms (erythema, edema, dryness and ulceration) (17). The scoring range indicators were 0 (none), 1 (mild), 2 (moderate) and 3 (severe). The specific symptom classification is shown in Table 1. The sum of the four symptom scores was calculated to assess SCORing of atopic dermatitis (SCORAD), with a maximum score of 12. In addition, the thickness of the skin lesion area and left ear of the mice were measured using electronic Vernier calipers (18). We got the skin images of the mice’s dorsal surface with a camera after anesthesia.

**TABLE 1:** AD score reference (maximum score: 12).

Score 1	Erythema
0	No erythema
1	Faintly visible punctate erythema
2	Patchy red papules
3	Dark red irregularly raised

**Score 2**	**Edema**

0	No edema
1	Slight edema
2	Localized edema with pitting exudate
3	Massive edema, more oozing, crusting

**Score 3**	**Dryness**

0	No dryness
1	Slight dryness of epidermis
2	Moderately dry epidermis with peeling
3	Severe dryness of epidermis with flaking

**Score 4**	**Ulceration**

0	No ulceration
1	Mild epidermal ulceration
2	Moderate epidermal ulceration
3	Severe infected epidermal ulcers

### Scoring of pruritic behavior

Pruritic behavior was observed. The pruritus score was defined by the duration of the pruritus behavior, that is, the time spent scratching and rubbing the skin of the ears and back with the limbs (16, 18, 19). On the last day of the experiment, the total duration of pruritic behavior of mice within 20 min was recorded with a high-definition camera. Scratching time less than 1.5 s was added 1 point each time; scratching time less than 3 s was added 2 points each time; scratching time more than 3 s was added 3 points each time; no scratching was scored 0 points. The total score was recorded as the pruritus score of mice.

### Pathological histological analysis of skin lesions

The dorsal skin of mice was collected and fixed in 10% neutral formalin for more than 48 h. The tissues were made into paraffin-embedded sections and stained with hematoxylin and eosin (H&E) and toluidine blue (TB). The histological changes of skin pathology on the dorsal skin of mice were observed under a light microscope at 100 × magnification. The thickness of the epidermis was measured, and the mast cell infiltration in the dermis was counted.

### Immunofluorescence staining analysis

The growth of PGP9.5 nerve fibers in the dorsal lesions of mice were analyzed by immunofluorescence staining of skin paraffin sections and observed under a fluorescent microscope. PGP9.5 antibody was purchased from Wuhan Servicebio Technology Co., Ltd. (China). Under ultraviolet laser, cell nuclei showed blue light after DAPI treatment, and PGP9.5 showed red light under the labeling of fluorescent secondary antibody. The fluorescence area and intensity were analyzed using Image J software.

### Calculation of spleen index

On the last day of the experiment, the spleens were weighed, and the splenic indices (spleen weight/body weight) were calculated.

### Immune cell counting

Blood was collected into tubes with EDTA, and the numbers of lymphocytes, eosinophils and basophils were counted using a hematology analyzer (Mindray).

### Enzyme-linked immunosorbent assay

The blood was collected and centrifuged at 3,000 r/min for 5 min, then the upper serum layer was separated and stored at –80°C. Serum levels of Ig E were determined using enzyme-linked immunosorbent assay (ELISA) kits (CUSABIO)^1^ according to the manufacturer’s instructions. Serum levels of Histamine (His) and Il-31 were measured by ELISA kit purchased from Shanghai Enzyme-linked Biotechnology Co., Ltd. (China).

### Real-time quantitative PCR

Total tissue RNA was extracted using the RNA isolater Total RNA Extraction Reagent kit, and RNA was reverse transcribed to cDNA using the HiScript III RT SuperMix for qPCR (+ gDNA wiper) reverse transcription kit. The reaction system was configured and performed according to the ChamQ Universal SYBR qPCR Master Mix kit. These kits were purchased from Nanjing Vazyme Biotech Co., Ltd. (China). The primers were synthesized by Beijing Tsingke Biotechnology Co., Ltd. (China). The primer sequences are listed in Table 2. The relative expression of target genes was analyzed by the 2-ΔΔCt method.

**TABLE 2:** Sequence of primers used for quantitative RT-PCR assay.

Gene	Forward primer (5′–3′)	Reverse primer (5′–3′)
β-Actin	TGCTGTCCCTGTATGCCTCTG	CTGTAGCCACGCTCGGTCA
Flg	CAATCCCACTCCAAACCATCTCCAG	GACTGTCCTCTGCCTCCTGATCC
Ifn-γ	CTCAAGTGGCATAGATGTGGAAG	TGACCTCAAACTTGGCAATACTC
Il-4	GGTCTCAACCCCCAGCTAGT	GCCGATGATCTCTCTCAAGTGAT
Lor	TTACTCCTCTCAGCAGACCAGTCAG	CCTCCACAGCTACCACCTCCTC
Tnf-α	CTGATGAGAGGGAGGCCATT	GCCTCTTCTCATTCCTGCTTG
Tslp	CTGCCATGATGAGGTGGTCTGAA	TCTGCTCACGAATTGTACTGTCCT

### Statistical analysis

The experimental data were statistically analyzed using GraphPad Prism 7.0 software. Data comparison between two groups was analyzed by *t*-test. Multiple data groups were compared using one-way ANOVA and Tukey’s multiple comparisons to analyze the variability between groups. *P* < 0.05 were considered statistically significant.

## Results

### Extraction rates of polysaccharides and total flavonoids from PO aqueous extract and PO ultrasound-assisted ethanol extract

**FIGURE 1:**
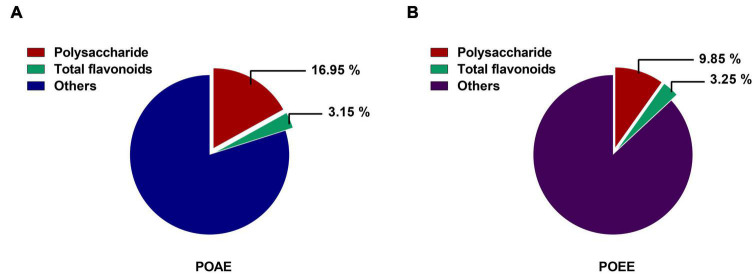
Polysaccharide and total flavonoid extraction rates of POAE and POEE in this study. **(A)** Polysaccharide and total flavonoid extraction rates of POAE. **(B)** Polysaccharide and total flavonoid extraction rates of POEE. Absorbance was measured at least 3 times and calculated based on the standard products (Glucose and Rutin). The data were expressed as mean.

Measurements of the phenol-sulfuric acid method showed that the polysaccharide extraction rates of POAE and POEE were 16.95% and 9.85%, respectively. The results of NaNO2-Al (NO3)3 colorimetric method showed that the extraction rate of total flavonoids in POAE and POEE were 3.15% and 3.25%, respectively (Figures 1A,B). The above results indicated that the extraction rate of PO total flavonoids was similar, while the extraction rate of polysaccharides of POAE was better than that of POEE.

### PO aqueous extract and PO ultrasound-assisted ethanol extract significantly alleviate 2,4-dinitrochlorobenzene -induced AD clinical symptoms in mice

**FIGURE 2:**
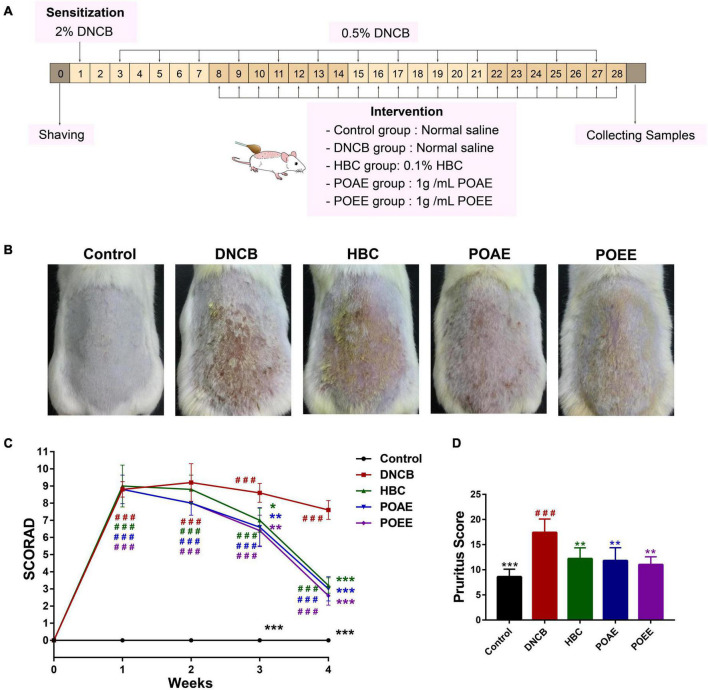
POAE and POEE significantly alleviate DNCB-induced AD clinical symptoms in mice. **(A)** Animal experiments. **(B)** Representative dorsal skin photographs of each group of mice. **(C)** SCORAD scores of each group of mice. **(D)** Pruritus scores of mice in each group. The data were expressed as mean ± SD (*n* = 5 per group). ^###^*P* < 0.001, vs. control groups; *P < 0.05, **P < 0.01, ***P < 0.001, vs. model (DNCB) groups.

We established a model of AD-like lesions in KM mice induced by DNCB and used HBC as a positive drug to investigate the role of POAE and POEE in AD mice (Figure 2A). Photographs of the dorsal skin of mice (Figure 2B) and SCORAD scores (Figure 2C) showed that POAE and POEE interventions significantly alleviated the clinical symptoms of dorsal skin. Pruritus lasted longer in the DNCB group, while the scratching behavior was strongly reduced after POAE and POEE interventions, comparable to that of the Control group (Figure 2D). The thickness of the dorsal skin and the left ear were effectively reduced after POAE and POEE treatments (Supplementary Figure 2). Generally, POAE and POEE interventions alleviate the symptoms of skin lesion and pruritus in AD mice.

### PO aqueous extract and PO ultrasound-assisted ethanol extractreduced the density of nerve fibers in the dorsal skin

**FIGURE 3:**
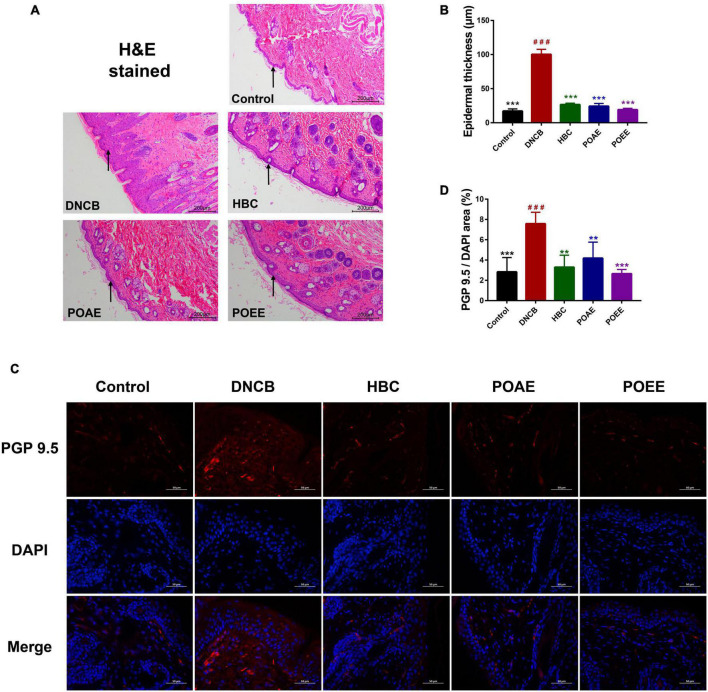
POAE and POEE reduced the density of nerve fibers in the dorsal skin. **(A)** Representative photographs of H&E staining of the dorsal skin of each group of mice (Scale bar: 200 μm). The black arrow indicates the epidermal layer of the skin. **(B)** Thickness of the epidermal layer of the dorsal skin of each group of mice (*n* = 3 per group). **(C)** Immunofluorescence staining of representative dorsal skin nerve fibers from each group of mice (Scale bar: 50 μm). The nuclei showed blue fluorescence after DAPI staining, and PGP9.5 showed red fluorescence under the labeling of fluorescent secondary antibody. **(D)** The ratio of PGP9.5/DAPI fluorescence area in each group of mice (*n* = 4 per group). The data were expressed as mean ± SD. ^###^*P* < 0.001, vs. control groups; **P < 0.01, ***P < 0.001, vs. DNCB (model) groups.

Next, we took a more in-depth observation of the dorsal skin of the mice. H&E-stained sections showed significant epidermal thickening in DNCB mice, whereas there was no significant difference between POAE and POEE groups and Control group (Figures 3A,B). Immunofluorescence-labeled skin sections showed that the fluorescence area of protein gene product 9.5 (PGP9.5) in the dorsal skin tissue of the DNCB group was obviously increased compared to Control group, indicating that repeated stimulation of the dorsal skin of mice by DNCB increased the density of nerve fibers. After treatment, the PGP9.5 fluorescence area was significantly reduced and nerve fiber density was decreased in the POAE and POEE groups compared with the DNCB group, which was remarkable (Figures 3C,D).

### PO aqueous extract and PO ultrasound-assisted ethanol extract reduced the number of immune cells and abnormal increase in serum Ig E in AD mice

**FIGURE 4:**
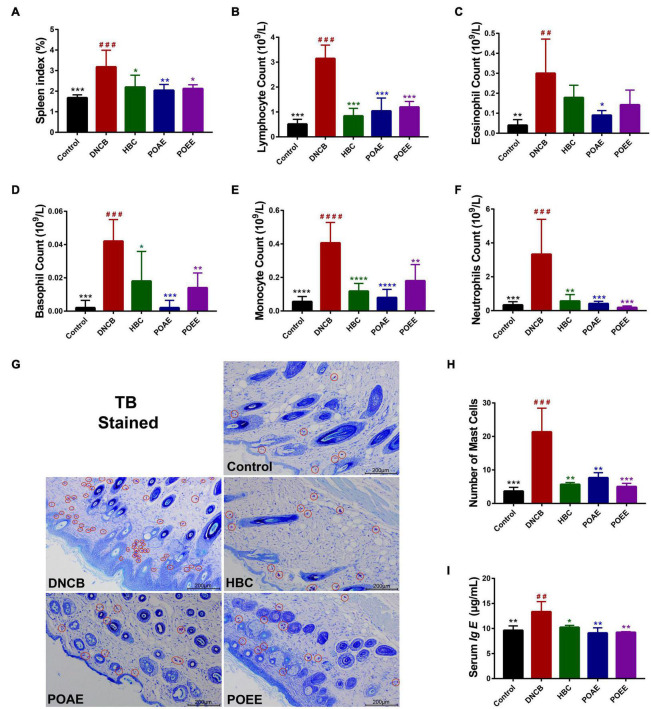
POAE and POEE reduced the number of immune cells and abnormal increase in serum Ig E in AD mice. **(A)** Spleen indices of mice in each group (*n* = 5 per group). **(B–F)** The number of lymphocytes, eosinophils, basophils, monocytes and neutrophils in each group of mice (*n* = 5 per group). **(G)** Pictures of representative dorsal skin TB staining of each group. Red circles mark some of the mast cells (Scale bar: 200 μm). **(H)** The number of mast cells in the dermis of each group (*n* = 3 per group). **(I)** Serum Ig E concentration of mice in each group (*n* = 3 per group). The data were expressed as mean ± SD. ^##^*P* < 0.01, ^###^*P* < 0.001, ^####^*P* < 0.0001, vs. control groups; *P < 0.05, **P < 0.01, ***P < 0.001, ****P < 0.0001, vs. model (DNCB) groups.

It is well-known that the spleen serves as the main organ of the body’s immunity, and an enlarged spleen indicates the activation of an immune response in metaplastic diseases (20). We found a significant increase in spleen index in DNCB-induced AD mice, while there was a marked decrease in spleen index in AD mice after POAE and POEE treatments (Figure 4A). The number of lymphocytes, eosinophils and basophils was significantly increased in the AD mice of the DNCB group, while POAE and POEE interventions caused different decreases in the number of these immune cells (Figures 4B–F). TB-stained sections of the dorsal skin showed severe mast cell infiltration in the dermis in the DNCB group, while mast cells were greatly reduced in the POAE and POEE groups (Figures 4G,H). In addition, serum Ig E levels were increased in the DNCB group compared to the control group, which were an important clinical indicator of AD; while serum Ig E was significantly decreased in the POAE and POEE groups compared to DNCB-induced mice (Figure 4I). The results showed that POAE and POEE reduced the number of immune cells and serum Ig E levels in mice.

### PO aqueous extract and PO ultrasound-assisted ethanol extract inhibit skin lesions and pruritus-associated cytokines in AD mice

**FIGURE 5:**
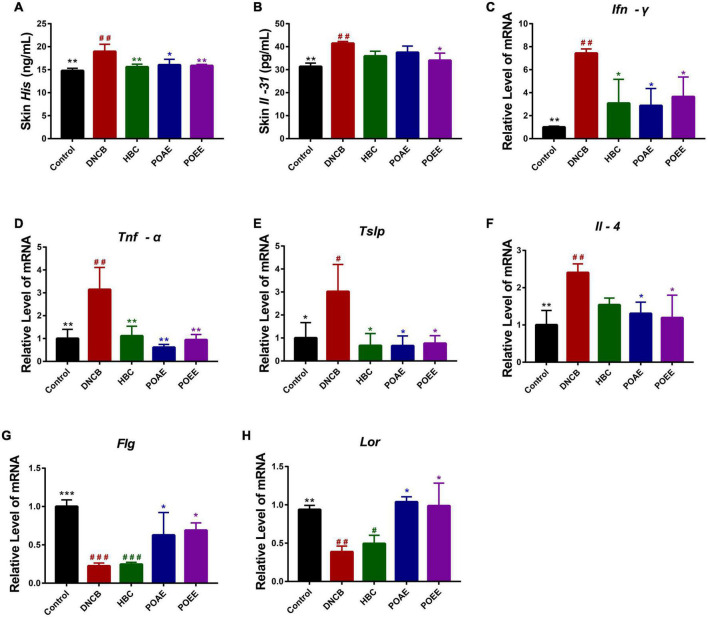
POAE and POEE inhibit skin lesions and pruritus-associated cytokines in AD mice. **(A)** Skin His concentrations in each group of mice. **(B)** Skin Il-31 concentrations in each group of mice. **(C–H)** Relative mRNA expression of Ifn-γ, Tnf-α, Tslp, Il-4, Flg, and Lor in each group of mice. The data were expressed as mean ± SD (*n* = 3 per group). ^#^*P* < 0.05, ^##^*P* < 0.01, ^###^*P* < 0.001, vs. control groups; *P < 0.05, **P < 0.01, ***P < 0.001, vs. model (DNCB) groups.

His is an important mediator of pruritus. Compared to the control group, DNCB induced an increase in skin His content in mice, while POAE and POEE were effective in reducing skin His concentration compared to the DNCB group (Figure 5A). In addition, the level of skin pruritic factor Il-31 was significantly increased, and it was lower in the POEE group (Figure 5B). The relative mRNA expression of inflammatory factors in skin lesions showed that interferon-γ (Ifn-γ), tumor necrosis factor-α (Tnf-α), thymic stromal lymphopoietin (Tslp) and Il-4 were significantly increased in DNCB-induced AD mice, and the relative mRNA expressions of Ifn-γ, Tnf-α, Tslp and Il-4 were significantly decreased after POAE and POEE treatments (Figures 5C–F). In addition, the relative mRNA expression of Filaggrin (Flg) and Loricrin (Lor) was impaired in AD mice, whereas POAE and POEE increased the mRNA levels of skin barrier proteins Flg and Lor (Figures 5G,H). The above results showed that POAE and POEE reduced the levels of skin His and Il-31 and down-regulated the mRNA levels of skin lesion-related factors, thereby suppressing the excessive immune response to AD and increasing the expression of skin barrier proteins.

## Discussion

PO has a high content of vitamins, minerals, omega-3 fatty acids, and is also rich in polysaccharides, flavonoids, alkaloids, terpenoids, and sterols. Thus, it is not only rich in nutritional value, but also has excellent pharmacological properties, and has great potential for use under sustainable development strategies (21). Recent reports indicate that PO has neuroprotective, antibacterial, antidiabetic, antioxidant, anti-inflammatory, anti-ulcer and anticancer activities, and plays an important role in alleviating symptoms such as inflammation, fever, headache, and insomnia (22, 23). Moreover, PO can regulate the gut microbiota, promote probiotics and inhibit pathogenic bacteria (24). Several studies have shown that PO has strong immunomodulatory effects, improving T-helper (Th) 1/Th2 and Th2/regulatory T (Treg) balance and reducing Ig E levels, thus exerting anti-inflammatory effects (25–27).

It has been reported that external application of a mixture of herbal extracts can alleviate skin inflammation and restore skin barrier integrity in AD mice (28). Studies show that Huanglian jiedu decoction can treat AD by modulating the antigen-presenting function of dendritic cells and attenuating T-lymphocyte activation, in turn exerting anti-inflammatory and anti-pruritic effects (29). Considering the abundant resources and numerous medicinal values of PO, we wondered whether external application of PO could alleviate AD-like skin lesions. Evidently, our studies showed that PO significantly alleviated the clinical symptoms and pathological changes of AD.

AD is a chronic recurrent skin disease characterized by eczematous, inflammatory, and severe pruritus. Previous study found that children with a family history of allergic disease or a parental history of AD were more likely to develop AD (30). Since AD is a heterogeneous disease with unique clinical manifestations in different age and ethnic groups, its pathogenesis is not yet completely clarified (31). In general, the pathogenesis of AD includes both genetic and external environmental factors of the organism. Imbalance of the body’s immune system, disruption of the skin barrier, induced infections and dysregulation of the skin microbiota, especially *Staphylococcus aureus*, also contribute to the pathogenesis of AD (32).

It is reported that Ig E and reactive T cells contribute to the pathophysiological development of AD (33). The increased level of Ig E is a sign of AD occurrence, as Ig E binds to numerous immune cells *via* high-affinity Ig E receptors, mediating the development of allergic inflammation (34). In this study, external application of POAE and POEE significantly reduced the serum Ig E levels in DNCB-induced AD mice. Moreover, lymphocytes, eosinophils and basophils were also reduced to varying degrees in the intervened AD mice. Eosinophils and mast cells mediate a large number of inflammatory molecules, including histamine, leukotrienes and interleukins, causing pruritus and mossy lesions in patients with AD (35). We found that the thickened epidermal layer and mast cell infiltration in AD-like lesioned mice were alleviated after PO intervention.

As mentioned above, pruritus is one of the most prominent and difficult features of AD. On the one hand, acute scratching serves as an adaptive defense against pruritogenic substances; on the other hand, chronic pruritus exacerbates the pruritic-scratching circulation, continuously leading to hair loss and skin damage (36). The sensation of pruritus is triggered by excitation of the nerve endings of the sensory nerves in the skin; furthermore, inflammatory mediators released by immune cells or skin cells may also sensitize sensory nerves and further exacerbate pruritic sensations (37). There are two main signals of pruritus in AD, histamine-dependent and non-histamine-dependent pathways, which seem completely independent, although the two systems are closely related (38). Histamine is an important pruritic mediator that induces a histamine-dependent pruritic response by binding to H1 or H4 histamine receptors and activating transient receptor potential vanilloid-1 (TRAV1) channels (39). Il-4 can rapidly amplify neuronal sensitization, including histamine-induced scratching behavior in response to various pruritogens. Non-histamine-dependent pruritic mechanisms involve numerous cytokines, neuropeptides, endogenous secretory factors, and sensitized nervous system (40). Abnormal increase in cutaneous nerve fibers is thought to be an important factor in causing pruritus symptoms in AD. In our study, skin nerve fiber density in AD mice was explored by using immunofluorescence to visualize protein gene product 9.5 (PGP9.5) ^+^ nerve fibers (41). The external application of PO reduced the skin nerve fiber density in AD mice, which was important in alleviating the exacerbation of skin lesions in AD mice caused by intense pruritus.

Abnormal immune responses in Th1/Th2 have been proposed to be critical in the development of AD. Most studies hold the view that Th2-type cells play a key role in acute AD and Tnf-α is required for antigen-specific Ig E production and induction of Th2-type cytokines and chemokines (42). Tslp is a key cytokine to promotes the Th2 immune response that is essential for the regulation of downstream Il-4/Il-13 and Th2 differentiation (43). Il-4, secreted by Th2 cells, is a cytokine closely related to the biological function of AD and continuously activate mast cells to produce more Ig E (44). It has been shown that elevated Th2 cytokines Il-4/Il-13 in AD lesions inhibit keratinocyte differentiation markers (FLG, LOR, keratin 1, and keratin 10) to impair skin barrier function (45). Accordingly, down-regulation of Il-4 expression is an important strategy for the treatment of AD. Transient receptor potential A1 (TRPA1) is mainly involved in non-histamine such as Il-31, Tslp-related pruritus, which is associated with pruritic transmission in the central nervous system (46). Il-31 is produced by activated T cells and is capable of inducing nerve fiber elongation (47). Il-31 can not only promote the release of pruritus-related neuropeptides, but also regulate the pathogenesis of AD by activating TRPV1 + /TRPA1 + sensory neurons (48). In addition, overexpression of Il-31 induced AD-like lesions, and comparison of TH1/TH2 cytokines suggested that Il-31 expression is associated with Il-4 and Il-13 but not Ifn-γ (49). The chronic phase of AD exhibits a local Th1 response, mainly associated with Ifn-γ. The down-regulation of Ifn-γ expression in patients after successful treatment of atopic dermatitis is remarkable (50). Mature Th1 cells secrete Ifn-γ and promote more Th1 cell differentiation. Dominance of Ifn-γ-producing T cells leads to chronicity of AD lesions and determines disease severity (51). Consistent with this, we observed that DNCB-induced AD-like lesion mice showed abnormal immune responses of Th1 and Th2, whereas external application of PO extract effectively reversed the significant elevation of serum Ig E, skin His, Il-31 levels and mRNA levels of Il-4, Tslp, Tnf-α and Ifn-γ, thereby alleviating AD-like atopic lesions and pruritus. These results suggest that PO is effective in ameliorating DNCB-induced AD in mice.

In conclusion, the main therapeutic targets in AD are Il-4/13, Il-5, Il-12/23, Il-17, Il-22, Il-31, Il-33, Tslp, and IgE (52). So far, what has been proven is that T cells are important drivers of AD and that the Th2 axis (especially Il-4/13, Il-31) contributes considerably to human AD. Therefore, most of the advanced AD drugs act on Th2 immunity, including the Il-4r antagonist Dupilumab, the biologic agent Dupixent targeting Il-4/13, the biologic agent Lebrikizumab targeting Il-13, and the humanized monoclonal anti-IL-31Rα antibody Nemolizumab (53). In addition, oral JAK inhibitors are considered promising drugs because they block a range of cytokine, growth factor, and hormone receptor signaling pathways (54). The oral JAK inhibitors Baricitinib and Abrocitinib are highly anticipated (55). But predictably, these emerging drugs would be extremely expensive. So, it would be an utmost blessing for AD patients to find cheaper yet effective drugs. Evidently, our study strongly supports that PO is a promising herb for the management of AD.

According to the results of this study, we found that PO can repair the skin barrier function (Flg and Lor) and also broadly modulate cytokines. PO reduces the release of Tnf-α from macrophages, Il-4, Il-31, and Tslp from the Th2 axis, and Ifn-γ from the Th1 axis, regulates the balance of immune cells, reduces the number of mast cells, lymphocytes, monocytes, neutrophils, eosinophils and basophils, reduces the secretion of His and Ig E, and thus alleviates allergic reactions, skin nerve fiber density and pruritus (38). We therefore speculate that the mechanism by which PO alleviates AD may be related to the inhibition of the release of Th1 and Th2 immune factors. PO plays an effective role in the management of AD lesion-like lesions by influencing the activity of immune cells through immunomodulatory effects. However, more detailed mechanisms need to be further investigated.

## Conclusion

In summary, this study provides an insight into the beneficial effects of PO on AD, which can help develop effective prevention or treatment strategies to combat AD and other inflammatory skin diseases. More detailed mechanistic, clinical and translational studies are needed to further substantiate the potential application of PO as a therapeutic agent for AD.

## Data availability statement

The raw data supporting the conclusions of this article will be made available by the authors, without undue reservation.

## Ethics statement

The animal study was reviewed and approved by the South China Agricultural University Experimental Animal Ethics Committee.

## Author contributions

W-JL and S-NG designed the overall research experiments. W-JL, J-YH, S-PL, and X-PG performed the experiments. J-YH and X-PG analyzed the data. W-JL and J-YH wrote the manuscript. S-NG, J-BS, and WM revised the manuscript. All authors contributed to the article and approved the submitted version.

## Footnotes

1
https://www.cusabio.com/
