# Supplementary material for: Portulaca oleracea L. extracts alleviate 2,4-dinitrochlorobenzene-induced atopic dermatitis in mice
Source: Front Nutr. 2022 Aug 16;9:986943. doi: 10.3389/fnut.2022.986943 (PMC9424637; doi:10.3389/fnut.2022.986943)
Supplement: Supplementary file 1 [file Data_Sheet_1.PDF]

Supplementary Material

1 Supplementary Figure 1

Comparison of the therapeutic effects of 0.5 g/mL POAE and 1 g/mL POAE in AD mice in our preliminary experiments.

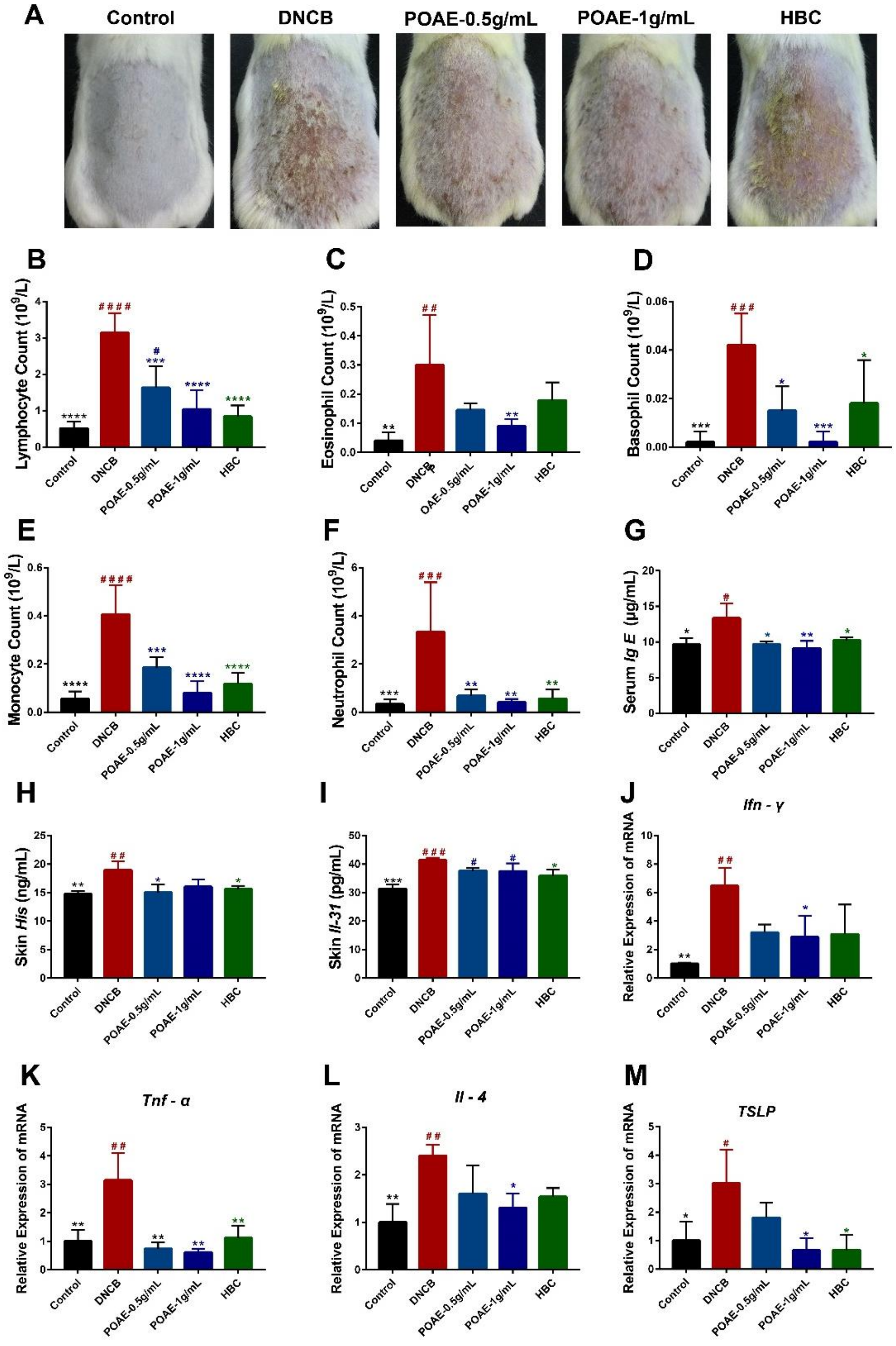

FIGURE S1 | Comparison of the therapeutic effects of 0.5 g/mL POAE and 1 g/mL POAE on AD mice. **(B)** Representative dorsal skin photographs of each group of mice. **(B-F)** The number of lymphocytes, eosinophils, basophils, monocytes and neutrophils in each group of mice (n=5 per group). **(G)** Serum Ig E concentration of mice in each group (n=3 per group). **(H)** Skin His concentrations in each group of mice. **(I)** Skin Il-31 concentrations in each group of mice. **(J-M)** Relative mRNA expression of Ifn-γ, Tnf-α, Il-4 and Tslp in each group of mice (n=3 per group). The data were expressed as mean ± SD. #: *P* <0.05, ##: *P* <0.01, ###: *P* <0.001, vs. control groups; *: *P* < 0.05, **: *P* < 0.01, ***: *P* < 0.001, vs. model (DNCB) groups.

2 Supplementary Figure 2

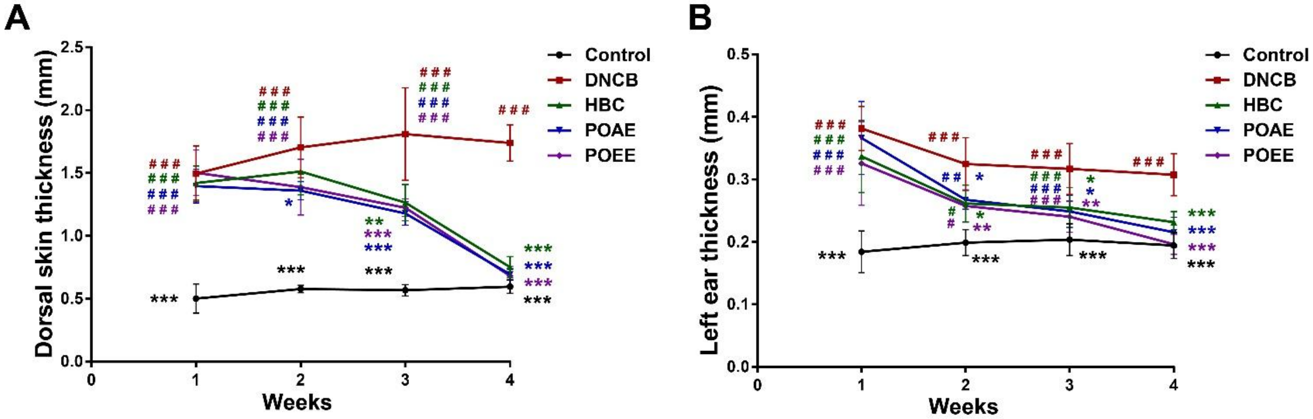

FIGURE S2 | POAE and POEE significantly alleviate DNCB-induced AD clinical symptoms in mice. **(A)** Dorsal skin thickness of mice in each group. **(B)** Left ear thickness of mice in each group. The data were expressed as mean ± SD (n=5 per group). #: *P* <0.05, ##: *P* <0.01, ###: *P* <0.001, vs. control groups; *: *P* < 0.05, **: *P* < 0.01, ***: *P* < 0.001, vs. model (DNCB) groups.
